# Diffuse aspiration bronchiolitis associated with oropharyngeal carcinoma: an underrecognized condition-a case report

**DOI:** 10.36416/1806-3756/e20260025

**Published:** 2026-08-13

**Authors:** Guilherme das Posses Bridi, Ellen Caroline Toledo do Nascimento, Eduardo Kaiser Ururahy Nunes Fonseca, Alexandre Franco Amaral, Bruno Guedes Baldi, Ronaldo Adib Kairalla

**Affiliations:** 1. Divisao de Pneumologia, Instituto do Coracao - InCor - Hospital das Clinicas - HCFMUSP - Faculdade de Medicina, Universidade de Sao Paulo, Sao Paulo (SP) Brasil.; 2. Departamento de Patologia, Faculdade de Medicina, Universidade de Sao Paulo, Sao Paulo (SP) Brasil.; 3. Instituto de Radiologia, Hospital das Clinicas - HCFMUSP - Faculdade de Medicina, Universidade de Sao Paulo, Sao Paulo (SP) Brasil.

## DEAR EDITOR:

Aspiration of oropharyngeal or gastric contents into the lower airways is an important and often underrecognized cause of pulmonary disease. The clinical spectrum of aspiration-related lung injury is broad, ranging from acute entities, such as aspiration pneumonitis and bacterial aspiration pneumonia, to chronic conditions associated with recurrent microaspiration, such as bronchiolocentric interstitial pneumonia and chronic small airway disease.^1^ ^-^ ^3^ While aspiration pneumonia is classically characterized by dependent airspace consolidation, repeated or occult aspiration may lead to more diffuse parenchymal and airway involvement, such as bronchiectasis, diffuse aspiration bronchiolitis (DAB), bronchiolitis obliterans syndrome and interstitial lung disease, including bronchiolocentric interstitial pneumonia.^4^ ^,^ ^5^ Increasing evidence indicates that aspiration diseases should be viewed not as a single entity, but as a heterogeneous group of syndromes with variable clinical, radiological, and pathological presentations, including different prognosis.^1^ ^,^ ^5^

Chronic aspiration has been implicated in the development or progression of interstitial lung abnormalities and fibrotic lung disease. Studies describing aspiration-related lung diseases have reported interstitial patterns associated with ongoing epithelial injury, persistent inflammation, and aberrant repair mechanisms, which could lead to progression or exacerbation of pre-existing lung diseases.^6^ ^,^ ^7^ Predisposing factors for aspiration include impaired consciousness (e.g., sedation, anesthesia), compromised airway protective reflexes, neurologic disorders, structural abnormalities of the upper aerodigestive tract, esophageal diseases, and gastroesophageal reflux disease.^5^ Despite growing recognition, DAB remains underreported, and its diagnosis is often delayed or overlooked.

**Figure 1:**
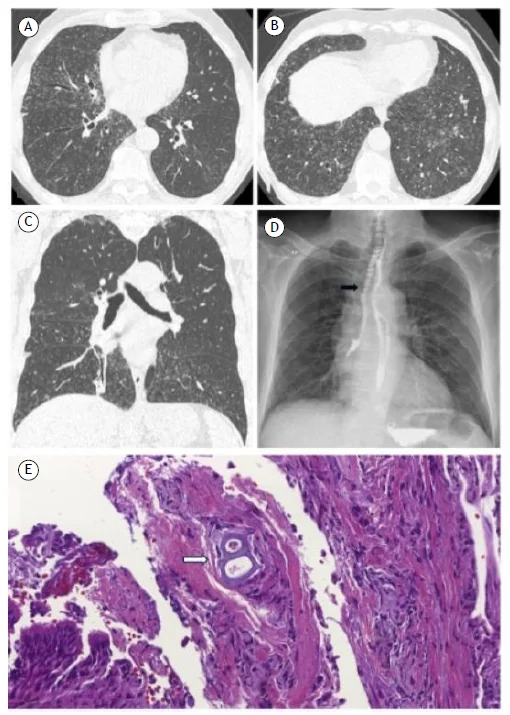
Chest CT scans (A-C) demonstrate findings that are consistent with diffuse aspiration bronchiolitis. Axial view (A and B) and coronal reconstruction (C) show numerous bilateral centrilobular micronodules with a tree-in-bud pattern, predominantly involving the lower lobes. Barium esophagogram (D) reveals an episode of direct bronchial aspiration, evidenced by contrast material visible within the central airways (black arrow). In E, transbronchial lung biopsy demonstrates a bronchiolocentric inflammatory infiltrate with areas of organizing pneumonia. Foreign material consistent with vegetable matter (white arrow) is identified within the airways, confirming chronic aspiration (H&E; magnification, ×200).

We report the case of a 74-year-old man with a one-year history of recurrent pneumonias. During that period, he developed fever, night sweats, and unintentional weight loss. He received multiple courses of oral antibiotics without significant clinical improvement. Diagnostic evaluation, including sputum analysis, bronchoalveolar lavage, and transbronchial lung biopsy ruled out tuberculosis and nontuberculous mycobacteriosis. Treatment with prolonged courses of prednisone and azithromycin showed minimal clinical improvement. The patient had a history of oropharyngeal carcinoma treated with chemotherapy and radiotherapy approximately 10 years before symptom onset, resulting in limited oral opening as a late sequela. He had no history of smoking and no clinical, laboratory, or imaging findings suggestive of autoimmune disease. Autoantibodies, including ANA, were negative. Lung auscultation revealed bilateral basal rhonchi and crackles, and peripheral oxygen saturation was 95% on room air at rest. Chest CT scan demonstrated multiple and bilateral centrilobular micronodules, occasionally exhibiting a tree-in-bud pattern, predominantly involving the bilateral lower lobes. A barium esophagogram was discontinued due to active aspiration of contrast media into the airways (Figure 1). A transbronchial lung biopsy revealed bronchiolocentric inflammatory infiltrate with areas of organizing pneumonia, and the presence of vegetable matter, confirming the diagnosis of DAB (Figure 1E). 

This case highlights the diagnostic challenge of DAB, which often presents with nonspecific respiratory and systemic symptoms and may mimic infectious or other inflammatory lung diseases. The predominant imaging findings-centrilobular micronodules and a tree-in-bud pattern with lower-lobe predominance-are characteristic but not pathognomonic and often lead to misdiagnosis as infectious or inflammatory bronchiolitis or mycobacterial disease. Other findings that may be identified include airway wall thickening, airspace consolidation, and, in a subset of patients, fibrosis.^2^ ^,^ ^3^ ^,^ ^5^ In this patient, the chronic course, lack of response to antimicrobial and anti-inflammatory therapies, and recurrent pneumonic episodes prompted further investigation. The incidental aspiration observed during the barium esophagogram reinforces the importance of considering swallowing dysfunction in patients with unexplained chronic bronchiolar disease.

Aspiration-related lung disease is particularly relevant in patients with a history of head and neck malignancies. Surgical interventions, chemotherapy, and radiotherapy can result in long-term structural and functional impairments of the oropharyngeal and esophageal phases of swallowing. Late sequelae such as dysphagia, impaired airway protection, and reduced oral opening significantly increase the risk of chronic microaspiration.^8^ Among patients with chronic occult aspiration, previous studies have shown a high prevalence of comorbidities such as gastroesophageal reflux disease, esophageal dysfunction, oropharyngeal or laryngeal dysfunction, hiatal hernia, obstructive sleep apnea, and obesity.^1^ ^,^ ^2^ Chronic or recurrent aspiration increases the risk of secondary infections due to persistent airway injury and impaired local defense mechanisms. In this setting, patients are particularly susceptible to bacterial superinfection and opportunistic pathogens, including mycobacteria.^2^ The spectrum of abnormalities related to chronic aspiration is broad and may range from lower-lobe bronchiectasis to bronchiolocentric interstitial pneumonias (Chart 1). Histopathological features in aspiration-related lung disease typically reveal a bronchiolocentric pattern of injury, characterized by chronic inflammatory infiltrates involving the airways. Common findings include organizing pneumonia, foreign-body giant cell reaction, and presence of exogenous material such as food particles or vegetable matter within bronchioles and alveolar spaces. Associated features may include interstitial fibrosis, foamy macrophages, and, in some cases, bronchiectasis, reflecting recurrent or chronic aspiration.^1^

**Chart 1:** Main diagnoses associated with chronic or occult pulmonary aspiration and key characteristics.

Aspiration-related bronchiectasis	- Bronchiectasis predominantly involving dependent lung regions - Frequent association with GERD, dysphagia or neurologic disease^1^
Diffuse aspiration bronchiolitis	- HRCT showing centrilobular nodules, tree-in-bud pattern -Associated with chronic microaspiration; histology may reveal bronchiolar inflammation with foreign material^1^ ^,^ ^5^
Bronchiolocentric interstitial pneumonia	- Inflammation and fibrosis centered around the small airways - HRCT typically demonstrates peribronchiolar GGOs, centrilobular nodules, and airway-centered reticulation - GERD, esophageal dysmotility, neuromuscular disorders, and impaired swallowing, and should be considered in the differential diagnosis^3^ ^,^ ^7^
Lipoid pneumonia	- Accumulation of lipids within the alveoli - Chronic aspiration or inhalation of oily substances (exogenous lipoid pneumonia) - Low-attenuation consolidation on HRCT, reflecting intrapulmonary fat^5^
Recurrent aspiration pneumonia	- Episodes of pneumonia affecting dependent lung segments (lower lobes and posterior upper lobes) - Fever, cough, and purulent sputum - Associated with impaired swallowing, altered consciousness, or significant GERD^4^

GERD: gastroesophageal reflux disease; and GGOs: ground glass opacities.

In patients with suspected occult pulmonary aspiration, a stepwise diagnostic approach may be helpful. The initial evaluation should begin with targeted clinical questions, focusing on symptoms like gastroesophageal reflux disease, dysphagia, chronic cough, or recurrent respiratory infections.^2^ ^,^ ^5^ A detailed assessment of comorbidities associated with aspiration risk, such as neurologic disease, neuromuscular disorders, prior stroke, esophageal disease, or head and neck conditions, should also be performed. If clinical suspicion persists, first-line investigations should include a speech and swallow evaluation to assess oropharyngeal dysfunction. In patients with suggestive symptoms or abnormal swallow assessment, further testing with a videofluoroscopic swallow study or barium esophagram may be considered.^1^ ^,^ ^4^

Management primarily focuses on addressing the underlying aspiration risk through multidisciplinary interventions, including swallowing rehabilitation, dietary modification, speech and language therapy, and, in selected cases, enteral feeding strategies. Pharmacological treatment plays a limited role and is generally reserved for managing complications such as secondary infection. The use of acid-suppressive therapy in patients with interstitial lung disease, such as idiopathic pulmonary fibrosis, has been debatable, but is currently recommended only for those with symptoms of gastroesophageal reflux disease.^4^ ^-^ ^6^ Early recognition of DAB is essential, as targeted management may prevent disease progression and reduce unnecessary exposure to antibiotics or immunosuppressive therapies. During follow-up, the patient was evaluated by a speech therapist, referred for gastrostomy, and oral feeding was contraindicated.
